# Development and National Validation of a Musculoskeletal Emergency Medicine Assessment Tool

**DOI:** 10.7759/cureus.57632

**Published:** 2024-04-04

**Authors:** William Denq, Alexander J Tomesch, Tyler Jackson, Allison D Lane, Anna Waterbrook

**Affiliations:** 1 Emergency Medicine, University of Arizona College of Medicine - Tucson, Tucson, USA; 2 Emergency Medicine/Sports Medicine, University of Nebraska Medical Center, Lincoln, USA; 3 Emergency Medicine, Hospital for Special Surgery, New York, USA; 4 Emergency Medicine/Sports Medicine, University of Arizona College of Medicine - Tucson, Tucson, USA

**Keywords:** orthopedic sports medicine, knowledge assessment, teaching in emergency medicine, medical resident education, musculoskeletal system

## Abstract

Introduction

Musculoskeletal (MSK) complaints and injuries are the fourth most common primary diagnosis in the emergency department in the United States (US). Despite the prevalence and economic impact on the US healthcare system, new emergency medicine (EM) residency graduates report feeling unprepared to treat MSK complaints. Currently, there are no reported means to assess MSK knowledge in EM resident physicians. The purpose of this study is to develop a validated and peer-reviewed multiple-choice assessment tool focused on MSK knowledge relevant to EM to allow us to better assess the knowledge of resident physicians.

Methods

A group of EM/Sports Medicine subject-matter experts (SMEs) created an initial list of the most important MSK topics in EM to generate a relevant question bank. The questions were validated by a different group of SMEs using a three-round modified Delphi method to obtain consensus on the importance of each question. Based on these results, the assessment was formed.

Results

From a list of 99 MSK topics, SMEs developed a final list of 37 MSK topics relevant to EM. Following round one, free-marginal kappa was 0.58, 95% CI [0.50, 0.66], with a moderate overall agreement of 71.95%. Following round two, the calculated free-marginal kappa increased to 0.88, 95% CI [0.83, 0.92], with an overall agreement of 91.79%. Using a five-point Likert scale, a threshold of an average score less than four was used to exclude questions in round three of validation and to create a final 50-question assessment tool.

Conclusion

Our proposed exam, titled Musculoskeletal Emergency Medicine Assessment Tool (MEAT), was successfully validated by experts in our field. It evaluates clinically important topics and offers a tool for assessing MSK knowledge in EM resident physicians. Future studies are needed to determine the feasibility of administering the tool and to establish a baseline score among different populations within the practicing field of EM.

## Introduction

Musculoskeletal (MSK) complaints and injuries account for 20% of presenting chief complaints and are the fourth most common primary diagnosis in United States (US) emergency departments (ED) [1]. Despite the prevalence, reports demonstrate a deficiency in MSK education in medical schools based on a validated MSK examination (FB-MSK) for graduating medical students [2-7] which may translate to incoming emergency medicine (EM) resident physicians. Comer et al. assessed EM attending and resident physicians at a Level 1 Trauma Center with the FB-MSK and reported that 35% of residents and 43% of attendings did not demonstrate proficiency [8]. In addition, 23% of participants reported dissatisfaction with their MSK education. In 2017, new EM residency graduates reported not feeling well prepared to care for MSK complaints [9].

Various national organizations provide guidelines and resources to aid EM residency programs with MSK educational interventions. A national EM task force from eight recognized organizations updates the Model of Clinical Practice of EM every two years [10]. This model describes EM clinical practice and is used by organizations, including residency programs, around the nation to structure core competencies and educational curricula including MSK education. However, the MSK section is limited, and the American Medical Society for Sports Medicine (AMSSM) recently recommended significant additions to the Model of Clinical Practice of EM and provided further curricular guidelines for MSK and Sports Medicine (SM) in EM residency [11]. The EM Council of Residency Directors (CORD) published an online SM toolkit offering modules, videos, and case presentations that offer a “plug and play” SM curriculum for EM residency programs [12].

Despite curricular guidelines and educational modalities being recommended to address a possible deficiency in MSK education of EM resident physicians, there is no documented assessment tool to determine if a deficiency exists. In addition, if a perceived or objective deficiency in MSK knowledge does exist, there are no prior reports of barriers to implementing an educational intervention to address it. We recently performed a national needs assessment of EM residencies for MSK education and identified time, interdepartmental relations, and funding as the top three barriers [13]. Most respondents believed their curriculum could be improved and they would utilize a standardized MSK assessment for EM residency training. Although validated MSK examinations exist, they were validated for medical students in 1998 (FB-MSK) and for medical trainees in a primary care context (MSK30) in 2019 [2,7]. Although both are useful, the FB-MSK was developed over two decades ago for medical students, and the MSK30 was developed for medical students entering the outpatient primary care field. As there is no relevant MSK assessment for EM resident physicians, the purpose of this study is to develop a validated and peer-reviewed MSK assessment tool to assess EM resident physician MSK knowledge.

## Materials and methods

A modified Delphi technique by means of three rounds of validation was completed by subject-matter experts (SMEs) across the US. In 2021, 16 SMEs practicing in nine different states were recruited by author consensus. These SMEs were all dual board-certified in EM and SM and felt to best represent the intersection of EM and MSK medicine. Although MSK SMEs exist in other specialties, the recruited demographic has unique clinical experience in both the ED and outpatient MSK clinics. These SMEs were separated into two groups, Group A and Group B, to perform topic and assessment development and assessment review and validation.

A subsection of Group A compiled a comprehensive list of MSK topics deemed most clinically relevant to EM based on the Model of Clinical Practice and AMSSM EM Curricular Guidelines. Group A reviewed the list and voted to combine, add, or remove topics. Any topic that had a greater than 50% vote was modified.

Group A assigned a blinded five-point Likert scale (one - not at all important, two - less important, three - somewhat important, four - more important, five - very important) to all topics and provided a recommended number of questions for the assessment tool. Any topic with a Likert score less than four was initially excluded.

Following these results, Group A discussed the results and reviewed the topic list. Several topics that were initially excluded were added to the list if the majority agreed. Group A then assigned a percentage weight to each topic recommended for the assessment tool. Based on the average weight, each member of the group was randomly assigned topics to develop questions and was assigned to independently review and modify questions written by others within Group A.

Group B provided feedback through the modified Delphi technique with three iterative rounds. In the first and second rounds, Group B was asked if each question should be included, modified, or deleted with the option to provide suggested edits. A free marginal kappa coefficient was calculated following each round. In the third round, a blinded five-point Likert scale (one - strongly disagree, two - disagree, three - neither agree nor disagree, four - agree, five - strongly agree) was utilized to determine if each question was appropriate and clinically relevant to EM.

This project was reviewed by the University of Arizona Institutional Review Board and approved as an exemption (2104683405, dated May 14, 2021).

## Results

A total of 99 MSK topics were determined to be clinically relevant to EM (Table 1).

**Table 1:** Initial list of musculoskeletal topics relevant to Emergency Medicine AP: anteroposterior; SC: sternoclavicular

Musculoskeletal topics
Achilles tendon rupture
Acromioclavicular joint separation
Acute spine sprain/strain
Amputation/replantation
Ankle reduction
AP compression of the pelvis
Apophyseal avulsion
Apophysitis
Arthrocentesis/joint block
Aseptic/Avascular necrosis
Avulsion injury
Axillary nerve - shoulder dislocation
Biceps tendon rupture
Bucket handle meniscus tear (locked knee)
Carpal tunnel syndrome
Cauda equina syndrome
Chronic/Degenerative back pain
Clavicle fracture
Coccyx fracture
Compartment syndrome
Computed tomography interpretation
Crystal arthropathies
Dermatome/myotome physical examination
Developmental dislocation of the hip
Disc disorders
Discitis
Dislocations
Elbow reduction
Epidural abscess/hematoma
Extremity fracture closed
Extremity fracture complicated
Extremity fracture open
Fasciitis
Felon/paronychia
Fight bite
Flail chest
Greenstick fracture
Hamstring rupture
High-pressure injection injury
Hip reductions
Inflammatory/infectious spondylopathies
Juvenile arthritis
Knee reduction
Laceration of tendons
Lateral compression
Lateral/medial epicondylitis
Ligament testing
Lisfranc injury
Lytic lesion/multiple myeloma/osteosarcoma
Muscle strains
Musculoskeletal examination
Musculoskeletal radiograph interpretation
Musculoskeletal ultrasound interpretation
Myositis
Nursemaids elbow
Osteoarthritis
Osteomyelitis
Patella reduction
Patellar/quadricep tendon rupture
Pathologic fracture
Perilunate/lunate dislocation/scapholunate dislocation
Peripheral neuropathy
Prosthetic dislocation
Radiculopathy/sciatica
Reactive arthritis
Rhabdomyolysis
Rheumatoid arthritis
Rib fracture
Rotator cuff tear
Sacroiliitis
Saline load test/traumatic arthrotomy
Salter-Harris classification
SC joint dislocation
Scapula fracture
Septic arthritis
Septic bursitis
Shoulder reductions
Slipped capital epiphysis
Soft tissue injuries to joints
Spinal shock
Spine dislocation/subluxation
Splinting
Spondylolisthesis/spondylolysis
Stable cervical spine fractures
Stable lumbar spine fractures
Stable thoracic spine fractures
Sternum fracture
Stress fracture
Synovitis
Tendinopathy
Tendonitis
Tenosynovitis
Thoracic outlet syndrome
Triceps tendon rupture
Unstable cervical spine fractures
Unstable lumbar spine fractures
Unstable thoracic spine fractures
Vertical shear
Xyphoid process fracture

By consensus, a list of 30 excluded topics that were mentioned in the AMSSM curricular guidelines was determined to be more related to EM/SM practice rather than EM MSK medicine (Table 2).

**Table 2:** List of excluded topics related to Emergency Medicine and Sports Medicine

Excluded topics
Bite wounds
Cardiovascular and thoracic procedures
Cardiovascular disorders
Chronic pain
Cutaneous disorders
Cutaneous infections
Dental trauma
Ear trauma
Endocrine
Environmental medicine
Eye trauma
Gangrene
Genitourinary trauma
Head trauma
Injury to nerve roots
Nail injuries
Nasal fracture
Orbital fracture
Palpitations
Paralysis
Paresthesia/Dysesthesia
Periarticular injuries
Peripheral nerve injury
Puncture wounds
Syncope/Near syncope
Tooth stabilization
Universal precautions
Wound closure
Wound management
Zygomaticomaxillary complex fracture

After a topic review with Group A, 16 changes were recommended to the included topics list. Following a majority vote, eight topics were modified, and one topic was removed (sternum fracture). A total of 98 topics were ranked by each blinded SME in Group A. Of the 98 topics, 37 topics were determined to have a Likert importance scale equal to or higher than four. After a final discussion with Group A, three topics that did not make the list were added by majority vote. Four topics, "dermatome/myotome", "musculoskeletal radiograph interpretation", "musculoskeletal ultrasound interpretation", and "splinting", were agreed to be concurrent themes assigned to topics in the final list of 40 topics (Table 3).

**Table 3:** Final list of musculoskeletal topics relevant to Emergency Medicine with a Likert importance score of greater than or equal to four, including percentage weight and number of questions assigned to each topic. concurrent theme - topics assigned to be concurrent with other topics in the final list; SC: sternoclavicular; SCFE: slipped capital femoral epiphyses; AVN: avascular necrosis

Final musculoskeletal topics	Likert score	Percentage weight	Number of questions assigned to topic
Achilles rupture	4.43	2.29%	1
Arthrocentesis	4.71	2.57%	1
Axillary nerve - shoulder dislocation	4.00	2.14%	1
Cauda Equina syndrome/Epidural abscess/hematoma	5.00	3.43%	2
Clavicle fracture	4.71	1.86%	1
Compartment syndrome	5.00	3.14%	2
Dermatome/Myotome	3.57	concurrent theme	concurrent theme
Discitis	4.00	1.29%	1
Elbow reduction	4.43	2.29%	1
Extremity fracture closed	5.00	7.29%	4
Extremity fracture open	5.00	3.00%	2
Felon/Paronychia	4.00	1.86%	1
Fight bite	4.29	1.57%	1
Flail chest/rib fracture	4.86	2.14%	1
High-pressure injection injury	4.57	2.00%	1
Hip reductions	4.86	2.43%	1
Lisfranc injury	4.00	1.86%	1
Musculoskeletal radiograph interpretation	5.00	concurrent theme	concurrent theme
Musculoskeletal ultrasound interpretation	3.71	concurrent theme	concurrent theme
Necrotizing fasciitis/Myositis	4.57	2.57%	1
Nursemaids elbow	4.14	2.29%	1
Osteomyelitis	4.43	2.29%	1
Patella reduction	4.57	2.00%	1
Patellar/Quad rupture	4.43	2.29%	1
Pediatric Hip (SCFE, AVN, Toxic synovitis)	4.57	3.57%	2
Pelvis fracture	4.43	3.14%	2
Perilunate/lunate dislocation/Scapholunate dislocation	4.29	2.29%	1
Rhabdomyolysis	4.57	1.86%	1
Salter-Harris classification	4.43	3.29%	2
SC joint dislocation	4.14	1.86%	1
Scapula fracture	4.14	1.43%	1
Septic arthritis/bursitis	5.00	3.43%	2
Shoulder/knee/ankle/prostethic reduction	5.00	5.29%	3
Splinting	5.00	concurrent theme	concurrent theme
Stable cervical spine fractures	4.00	1.71%	1
Tendon laceration	4.57	1.86%	1
Tenosynovitis	4.14	2.00%	1
Traumatic arthrotomy/saline load test	3.71	1.57%	1
Unstable cervical spine fractures	4.71	3.71%	2
Unstable thoracic/lumbar spine fractures	4.71	3.14%	2

After question and assessment development, free-marginal kappa was calculated in rounds one and two until an overall agreement of 91.79% was achieved (Table 4).

**Table 4:** Calculated free-marginal kappa for rounds one and two of assessment development

Round	Free-marginal kappa	95% confidence interval	Overall agreement
One	0.58	0.50, 0.66	71.95%
Two	0.88	0.83, 0.92	91.79%

In round three of the modified Delphi method, responses were compiled from all SMEs and an average score was calculated for each question. A final threshold of an average score of less than four was decided upon to exclude questions in round three of validation. After group discussion and consensus, the validated 50-question assessment tool was created (see Appendices).

## Discussion

Currently, there is no established gold standard for training and educating EM resident physicians in MSK knowledge. To our knowledge, this 50-question Musculoskeletal Emergency Medicine Assessment Tool (MEAT) is a novel assessment for EM developed through a rigorous process by an expert cohort. Through MEAT, EM residency programs can assess relevant MSK knowledge and utilize the results to address any notable deficiencies. The efficacy of residency-wide interventions such as an orthopedic or SM rotation or an MSK block in grand rounds can be evaluated through MEAT. We previously reported on pre- and post-orthopedic rotation MSK knowledge acquisition through FB-MSK assessment but can replace the FB-MSK with MEAT to compare results [14,15]. Incoming resident physicians can be evaluated as a baseline and then retested upon graduation to determine if MSK knowledge was gained. Evaluators can administer MEAT through online or paper distribution and assign a one-point score to each question for a total score of 50. Programs can also reference the topic list MEAT is based on to create their own questions or educational interventions. This should allow for the development of more individualized and robust curriculums for EM residents.

EM residency programs have a significant amount of physician tasks, medical knowledge, patient care, and procedural skills to learn over the course of a three- or four-year residency. Although MSK pathologies may not constitute a majority of critical conditions, one in five patients presenting to an ED in the US will have an MSK-related chief complaint [1]. The ability to differentiate “sick” from “not sick” starts with a fundamental understanding of a domain, such as MSK. Family medicine identified this deficit in the outpatient setting and developed the MSK30, an assessment for graduating medical students and primary care resident physicians [7]. With MEAT, EM educators and clinicians may have a starting point to identify deficiencies in MSK knowledge. Once deficiencies are identified, residency-wide or individualized solutions can be employed from published resources [11,14]. Focusing solutions on identified deficiencies may save time in the residency curriculum, which was identified as a barrier to implementing the MSK curriculum [13].

The next step is to determine the feasibility of administering MEAT to EM resident physicians. Our goal is to assess this tool in a single and then multi-institutional study. Based on these results, we hope to establish a baseline score among EM resident physicians entering residency to help determine the success of MSK educational interventions. Subsequently, comparing MEAT to clinically relevant endpoints would allow for potential future iterations and improvements to the assessment tool.

Given MEAT was developed to assess MSK knowledge in EM resident physicians, it is possible to extrapolate and utilize this tool for board-certified EM physicians. The EM-relevant MSK topics chosen for MEAT would not change in clinical practice from resident to attending physician. It may even be used to reassess MSK knowledge after a period following board certification.

There are several limitations to this study. Although we recruited from multiple states across the country to incorporate different training backgrounds and practice patterns, we did not recruit a specific breakdown of SMEs (e.g., academic versus community physicians) nor did we randomly select SMEs from a prepopulated list. Although the decision to recruit dual-board certified EM/SM physicians was felt to be the most relevant to EM MSK knowledge due to clinical experience, other specialty SMEs could be involved in future studies. The top 50 list of topics is still expert opinion, and therefore subject to bias and susceptible to future emerging topics. While SMEs were blinded to each other's responses, it is possible that they communicated with each other about their responses or discussed the study with an outside party.

## Conclusions

Our proposed assessment, the MEAT, fills a gap in the current EM MSK curriculum. It evaluates clinically important topics and offers a specific tool to assess clinical MSK knowledge in EM. Educators may be able to use this tool to develop further educational interventions. Future studies are needed to assess the feasibility of administration and establish a baseline score of MSK knowledge for different subject populations across EM practice.

## Figures and Tables

**Figure 1:**
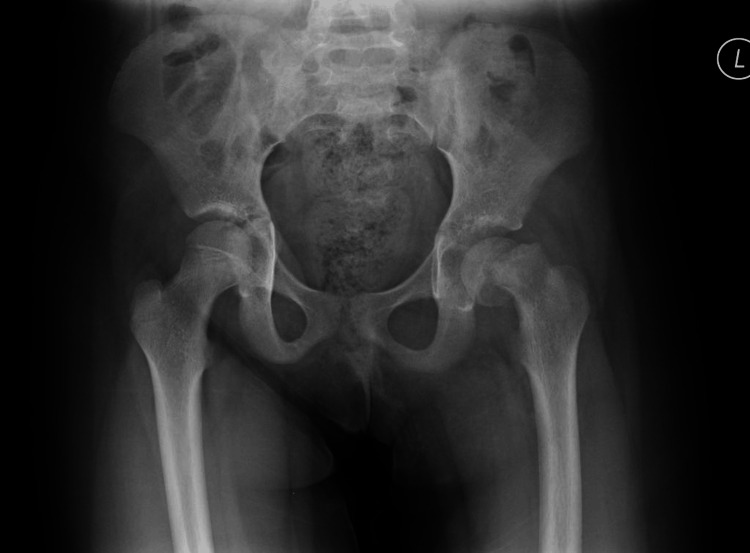
Anteroposterior radiograph of the pelvis Case courtesy of Hani Makky Al Salam, Radiopaedia.org

**Figure 2:**
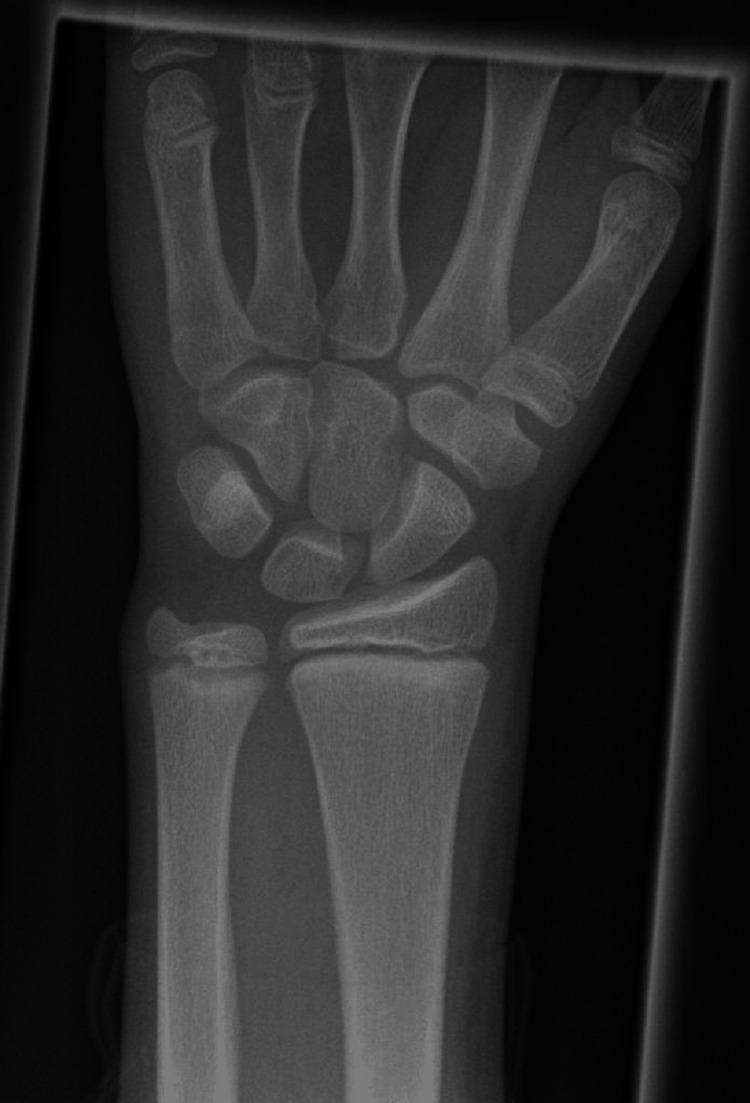
Anteroposterior radiograph of the wrist Image courtesy of Ian Bickle, Radiopaedia.org

**Figure 3:**
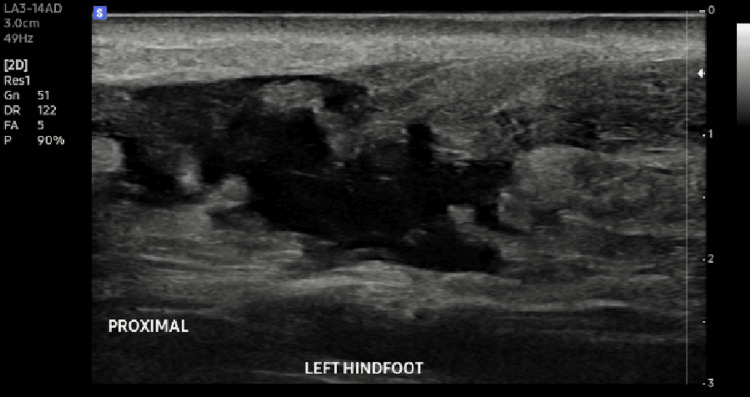
Longitudinal ultrasound view of the left hindfoot Image courtesy of Matthew Negaard

**Figure 4:**
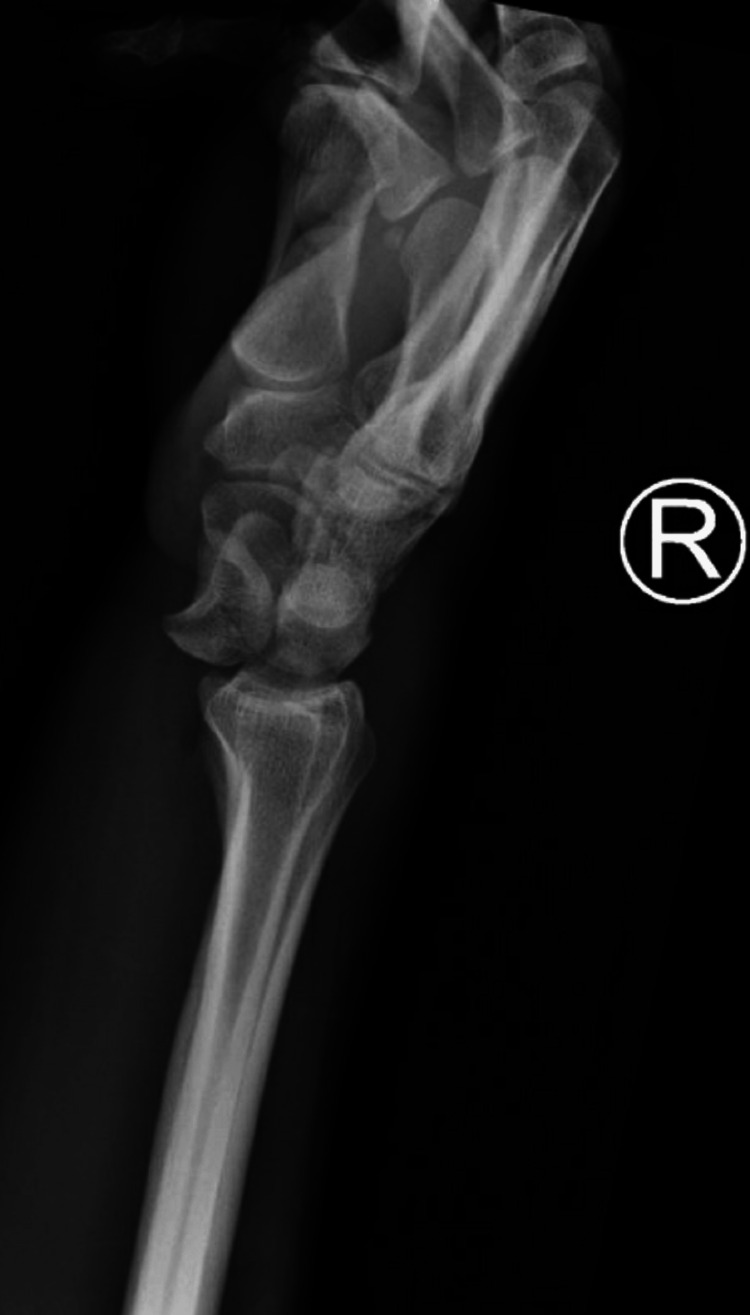
Lateral radiograph of the wrist Image courtesy of Will Denq

**Figure 5:**
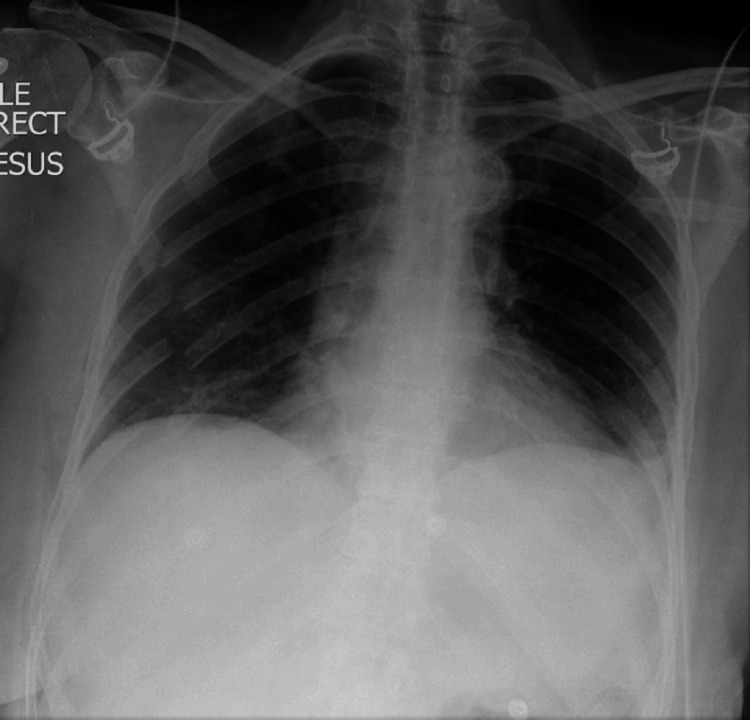
Anteroposterior radiograph of the chest Case courtesy of Ian Bickle, Radiopaedia.org

**Figure 6:**
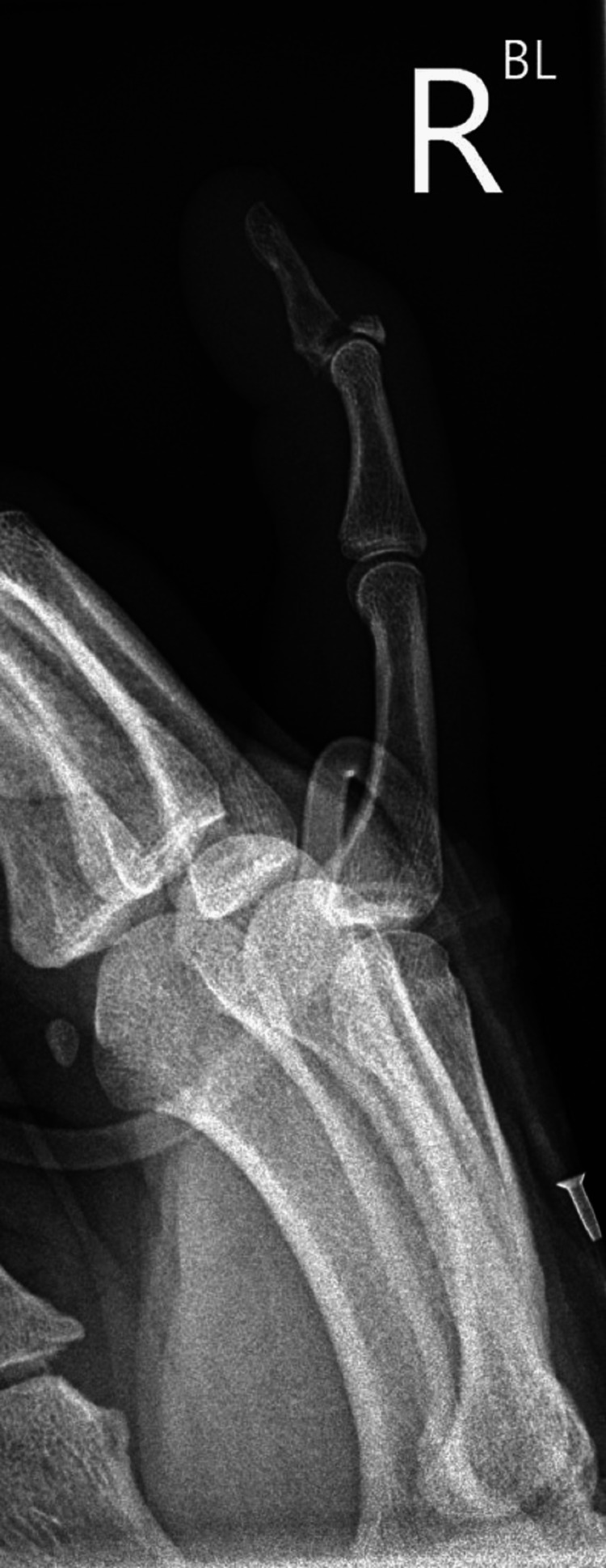
Lateral radiograph of the digit Case courtesy of Andrew Taylor, Radiopaedia.org

**Figure 7:**
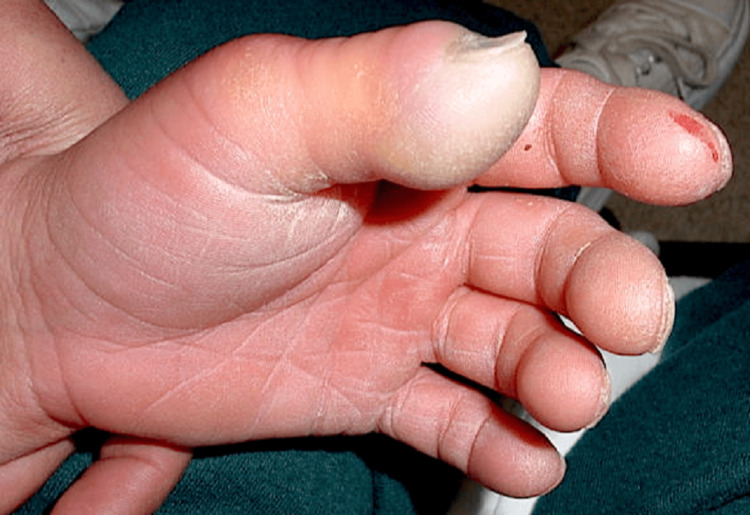
Photo of the affected digit Image courtesy of Adam Rosh, Rosh Review

**Figure 8:**
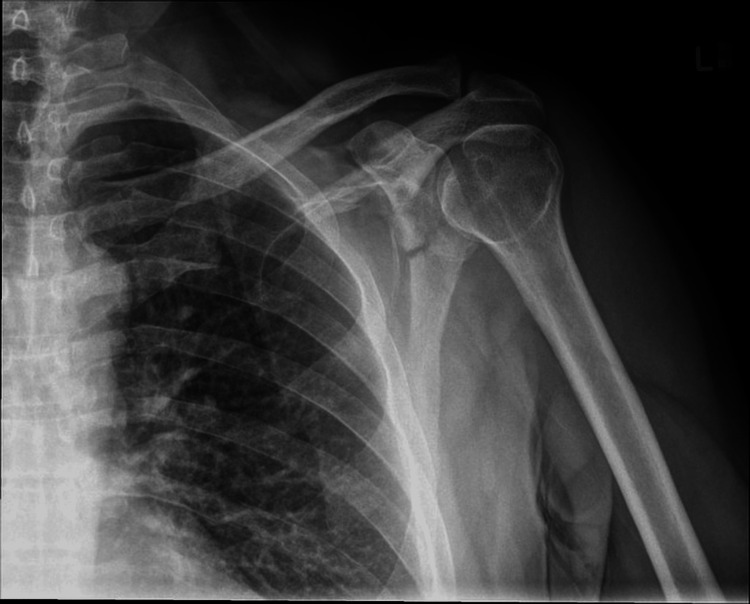
Anteroposterior radiograph of the shoulder Case courtesy of Henry Knipe, Radiopaedia.org

## Appendices

**Table 5:** Musculoskeletal Emergency Medicine Assessment Tool

Question	Answer A	Answer B	Answer C	Answer D	Figure
1. A 74-year-old male presents with a distal radius fracture. You attempt to reduce it in the emergency department. Which of the following best describes your reduction technique?	Apply an axial load, restore alignment, then recreate the mechanism of injury	Apply an axial load, recreate the mechanism of injury, then restore alignment	Apply traction, recreate the mechanism of injury, then restore alignment	Apply traction, restore alignment, then recreate the mechanism of injury	
2. You obtain an elbow x-ray on a pediatric patient and are concerned about a supracondylar fracture. There is no posterior fat pad sign on the lateral view. What additional radiographic feature helps reduce the likelihood of a supracondylar fracture?	Anterior humeral line should intersect the middle third of the capitellum	Anterior humeral line should intersect the posterior third of the capitellum	Posterior humeral line should intersect the middle third of the capitellum	Posterior humeral line should intersect the posterior third of the capitellum	
3. A 22-year-old college student presents to your emergency department for evaluation. He has a fracture of the 5th metacarpal neck on x-ray. What is the correct name and splint for this injury?	Radial Gutter splint	Sugar Tong splint	Thumb spica splint	Ulnar gutter splint	
4. You are evaluating a 22-year-old patient with a fall on an outstretched hand. The patient has anatomical snuff box tenderness. Radiographs are negative. Which management step is most appropriate?	Compression dressing	Sugar tong splint	Thumb spica splint	Ulnar gutter splint	
5. You are evaluating a middle-aged male with right shoulder pain. He states he fell backwards on a ladder and felt a pop. You are suspicious of glenohumeral dislocation. Which plain film view would be most appropriate to accurately diagnose an anterior dislocation?	Anterior-posterior view	Axillary view	Internal rotation view	Zanca view	
6. You are evaluating a 54-year-old male with a significant knee injury after a motorcycle crash. He reports he had anterior translation of the tibia prior to it “popping back in”. On exam, he has a 1+ DP and PT pulse, decreased compared to the contralateral side, what is the most appropriate next step in management?	If the ankle-brachial index is less than 0.9, discharge home with urgent outpatient orthopedics follow-up	Perform computed tomography angiography, consult Orthopedics, and consult Vascular surgery if vascular injury is found	Place in knee immobilizer, discharge home with urgent outpatient orthopedics follow-up	Serial exams for 2 hours, if neurovascular exam remains the same, discharge with urgent outpatient orthopedics follow-up	
7. A patient presents with right ankle pain after a significant fall. He is found to have a posterior ankle dislocation. What is the most appropriate technique used to reduce these injuries?	Extend the knee, dorsi-flex the ankle, apply axial traction on the foot and then simultaneously posterior pressure on the calcaneus	Extend the knee, plantar-flex the ankle, apply axial traction on the foot and then simultaneously apply lateral pressure on the calcaneus	Flex the knee, plantar-flex the ankle, apply axial traction on the foot and then simultaneously apply anterior pressure on the calcaneus	Flex the knee, plantar-flex the ankle, apply axial traction on the foot and simultaneously apply medial pressure on the calcaneus	
8. A 22-year-old male presents to the ED after falling and striking his head while intoxicated. There is concern for cervical spine injury. Which of the following is considered a stable cervical fracture?	Bilateral facet dislocation	Flexion teardrop fracture	Jefferson fracture	Wedge fracture	
9. In addition to your usual Anterior-Posterior and Lateral views, what other x-ray view can be most useful when evaluating for C1 burst fracture?	Extension View	Flexion View	Odontoid View	PA oblique view	
10. You are evaluating a 23-month-old female who presents with a limp on weight-bearing. Her mom states she has been limping for a few days. She denies any trauma, but endorses recent upper respiratory infection symptoms. She is currently afebrile. Which of the following diagnoses is most likely?	Septic arthritis	Slipped capital femoral epiphysis	Toddler’s fracture (tibial shaft fracture)	Transient synovitis of the hip	
11. A 13-year-old obese male presents to the ED with left hip and knee pain progressing over the last month. Radiographs obtained are shown below (Figure 1). What is the most likely diagnosis? Figure 1: Case courtesy of Hani Makky Al Salam, Radiopaedia.org	Legg-Calves-Perthes Disease (LCPD)	Lesser trochanter avulsion fracture	Normal variant	Slipped Capital Femoral Epiphyses (SCFE)	Figure 1
12. A 60-year-old female presents with acute on chronic low back pain. She has a history of IV heroin use. Lately, she has been having fevers, fatigue, and difficulty walking. Vital signs are T: 39, BP: 90/55, P: 122, R: 26. What is the best initial step?	Intravenous antibiotics	Lumbar puncture	Magnetic Resonance Imaging	Neurosurgical consultation	
13. A 50-year-old male presents with acute on chronic low back pain. He now feels pain down his both legs and they feel weak. Bedside ultrasound shows a post-void residual of 300 mL. What is the most appropriate disposition?	Emergent neurosurgery consult for surgical decompression	Follow up with neurosurgery for outpatient surgical intervention	Intravenous antibiotics and admission	Outpatient physical therapy	
14. A patient presents to the ED for a hot and swollen ankle. On examination, they have pain with passive range of motion. You perform an arthrocentesis. The results of the fluid analysis show 120,000 WBC, 96% neutrophils, and negatively birefringent crystals in needle shapes. What is the diagnosis and the next step in management?	Gout, Discharge	Osteoarthritis, Ibuprofen	Pseudogout, Prednisone	Septic arthritis, consult orthopedics	
15. Patient presents to the emergency department with swelling over the left olecranon. They have no pain with pronation/supination of the elbow. The overlying skin is unremarkable with no erythema or increased warmth. What is the next best step?	I&D for concern of abscess	Perform a diagnostic tap to rule out septic arthritis	Perform a therapeutic tap to drain all fluid from this collection	Recommend compression and anti-inflammatory treatment	
16. You are evaluating an 8-year-old athlete who rolled his ankle playing basketball. On exam, there is exquisite tenderness to the lateral malleolus. Radiographs show a fracture line only through the epiphysis and does not cross the physis. Which Salter-Harris classification would this child have?	Salter-Harris I	Salter-Harris II	Salter-Harris III	Salter-Harris IV	
17. A 10-year-old female presents with wrist pain following a FOOSH off her bicycle. On exam there is swelling and tenderness over the physis of the distal radius. There is no tenderness to the anatomic snuff box. Radiograph is shown below (Figure 2). What would be the suspected diagnosis and treatment? Figure 2: Image courtesy of Ian Bickle, Radiopaedia.org	Salter-Harris I fracture; Splint	Salter-Harris V fracture; Splint	Scaphoid fracture; Splint	Sprain; ACE wrap	Figure 2
18. A 33-year-old male presents to your ED with leg pain. He was seen yesterday and diagnosed with a tibia fracture, placed in a splint and discharged. He returns due to severe pain. Even after removing the splint he is in severe pain at rest, pain with passive stretch, decreased sensation, and his anterior compartment is tense. Your nearest hospital with orthopedics is 6 hours away. Which would be the most appropriate treatment plan?	Elevate the leg to decrease swelling, pain control with regional block, obtain compartment pressures	Intravenous pain medication, consider fasciotomy, transfer to hospital with orthopedics	Keep the leg in a dependent position to increase perfusion, pain control, obtain compartment pressures	Pain control with regional block, transfer to hospital with orthopedics	
19. A patient presents to the ED after a long bone fracture. In which of the following presentations would you be most concerned for acute compartment syndrome?	A patient with a crush injury to the forearm with a delta pressure of 35 mmHg	A patient with a femur fracture and diastolic blood pressure of 50 and intra-compartment pressure of 25 mmHg	A patient with fibula fracture and delta pressure of 50 mmHg	A patient with a tibia fracture with anterior compartment pressure of 20 mmHg	
20. A patient presents after being struck by a vehicle while crossing the street. He is reporting severe back pain. Which of the following thoracic spine fractures would most likely be unstable?	Burst fracture	Clay-Shoveler fracture	Compression fracture	Transverse process fracture	
21. You are evaluating a 33-year-old female in the trauma bay status post high speed MVC. She has multiple spinal fractures. Which of the following fractures should increase your index of concern for concomitant intra-abdominal injury?	Burst fracture	Chance fracture	Clay-Shoveler fracture	Compression fracture	
22. A patient presents to the Emergency Department after a motor vehicle accident. She reports abdominal and pelvic pain. What pelvic fracture is considered unstable?	Avulsion fracture of the anterior iliac spine	Iliac wing fracture	Superior and inferior pubic ramus fracture	Vertical shear fracture	
23. A patient presents to the Emergency Department after a motor vehicle accident. He reports abdominal and pelvic pain. His blood pressure is 86/55. Examination reveals gross pelvic instability. Where should a pelvic binder be centered over?	Greater trochanters	Inferior iliac spine	Iliac crest	Superior iliac spine	
24. A patient presents to the Emergency Department after a tractor roll-over. He has a tibia fracture with a 15 cm wound with significant tissue loss, that will require tissue flap to cover. The wound is grossly contaminated with dirt. What is the most appropriate antibiotic regimen?	Cefazolin and Ciprofloxacin	Cefazolin, Penicillin G, and Gentamicin	Ceftriaxone and Clindamycin	Ceftriaxone and Gentamicin	
25. A 52-year-old male with an open tibia fracture presents to the ED. The tibial fracture is protruding through the skin. How should the open fracture be managed?	Consult orthopedics for surgical intervention	Place a wet dressing, consult orthopedics for surgical intervention	Start IV antibiotics, reduce fracture, place a splint, consult orthopedics for surgical intervention	Wash out wound in the ED, reduce the fracture, place a splint, close the wound, and discharge	
26. A patient presents to the ED after a fall off their bicycle. Workup shows midshaft humeral fracture. The patient has an associated neurological deficit, what examination technique would show the expected deficit?	Finger abduction	Finger flexion	Thumb adduction	Wrist extension	
27. You are evaluating a patient with atraumatic knee pain and no systemic symptoms. He has normal vital signs. He has an effusion and history of significant alcohol use. Which of the following fluid analysis is expected?	5,000 WBC, neutrophils 60%, positively birefringent rhomboid shaped crystals	5,000 WBC, neutrophils 75%, no crystals	15,000 WBC, neutrophils 50%, negatively birefringent needle shaped crystals	100,000 WBC, neutrophils 97%, no crystals	
28. A 40-year old male history of IV substance abuse presents with left calf tenderness and swelling. He reports fatigue and subjective fevers. Temperature 101.4 F. Heart rate is 98. WBC 12.5, ESR 65, CRP 40. CK is within normal limits. A radiograph of the tibia/fibula demonstrates soft tissue swelling and gas formation. What is your next step?	Emergently consult for surgical evaluation	Obtain an MRI of the lower extremity	Obtain a CT of the lower extremity	Perform a finger test	
29. A patient presents to the ED 3 weeks after total hip arthroplasty, unable to bear weight on the leg. X-ray shows anterior hip dislocation. What additional diagnostic testing should be performed in addition to reducing the hip and consulting orthopedic surgery?	Computed tomography of the hip	Laboratory workup to rule out infection	Magnetic resonance imaging of the hip	Ultrasound of the hip	
30. A patient presents with a chronic foot ulcer. Which imaging modality is most sensitive and specific for identifying acute osteomyelitis?	Computed tomography	Magnetic resonance imaging	Plain film radiographs	Ultrasound	
31. You are evaluating a 44-year-old male who had sudden ankle pain and felt a pop while playing basketball. You perform an ultrasound of the posterior ankle that looks like the below (Figure 3), what is the diagnosis? Figure 3: Image courtesy of Matthew Negaard	Achilles tendon rupture	Calcaneal spur	Ganglion cyst	Gastrocnemius strain	Figure 3
32. A 54-year-old male presents with knee pain. He states that he was playing basketball when he felt a sudden knee pain after jumping and landing. He is unable to extend his knee against gravity. Given the most likely diagnosis, what is the most appropriate form of immobilization for this patient?	Hinged knee brace	Knee immobilizer	Stirrup splint	Tall walking boot	
33. You evaluate a 42-year-old male roofer with elbow pain status post fall off the roof. After confirming he is neurovascularly intact, you obtain X-rays that reveal a posterior elbow dislocation. You successfully reduce it. What immobilization method is most appropriate for a reduced, posterior elbow dislocation?	Posterior long arm splint	Shoulder immobilizer	Shoulder sling	Sugar tong	
34. A patient presents to the ED after a FOOSH injury. X-ray is obtained and shown below (Figure 4). What is the most appropriate next step in management? Figure 4: Image courtesy of Will Denq	Closed reduction and splinting with close hand surgery follow-up	Magnetic resonance imaging of the wrist	Short arm cast	Thumb spica splint with repeat x-rays in 1 week	Figure 4
35. You have a 3-year-old in your pediatric emergency department who is not using their arm. You suspect a nursemaid's elbow. What is the specific injury associated with this diagnosis?	Apophysitis of the olecranon	Dislocation of the radial head	Salter-Harris 1 fracture	Subluxation of the radial head	
36. A 21-year-old male presents with chest pain after he was struck by a speeding sedan. On examination, you notice paradoxical movement of the right chest wall. Vital signs are significant for SpO2: 89%. Point of care ultrasound shows positive slide sign in all fields. There are no open wounds of the chest wall noted. Chest radiograph below (Figure 5). What is your immediate next step? Figure 5: Case courtesy of Ian Bickle, Radiopaedia.org	Perform a CT of the chest	Place a chest tube	Place the patient on supplemental oxygen	Place a temporary splint over the injured area to assist with breathing	Figure 5
37. A 33-year-old prison inmate presents with right shoulder pain. He is diagnosed with a shoulder dislocation, which is subsequently reduced. What nerve is most commonly injured in an anterior shoulder dislocation?	Axillary	Median	Radial	Ulnar	
38. A 25-year-old industrial painter presents to the ED at his boss’s behest after accidentally running the tip of his index finger under a paint sprayer. He denies any pain or discomfort and has full range of motion of his finger. What is the most appropriate next step in management?	Bedside I&D with digital block	Discharge with oral antibiotics and outpatient follow-up with PCP	Intravenous antibiotics and observation x 24 hours	Urgent hand surgery consultation	
39. You are evaluating a 17-year-old football player with right knee pain and an obvious deformity following a collision. You suspect a lateral patellar dislocation. Which of the following best describes the reduction technique?	Gently extend knee while applying laterally-directed pressure to patella	Gently extend knee while applying medially-directed pressure to patella	Gently flex knee while applying laterally-directed pressure to patella	Gently flex knee while applying medially-directed pressure to patella	
40. You are evaluating a 34-year-old patient with hand pain. They state that they had a puncture wound while swimming in the ocean. They present to the ED with worsening pain and you are suspicious of flexor tenosynovitis. Which of the following correctly identifies one of the Kanavel signs?	Finger is held in slight extension	Localized swelling of the pulp of the fingertip	Pain with passive flexion	Tenderness over the flexor tendon	
41. A patient presents to the ED after a bicycle accident where he landed on the left shoulder. His x-ray shows a mid-clavicle fracture with 1 cm of displacement and no skin tenting. What is the appropriate disposition?	Attempt closed reduction, place in arm immobilizer, non-weight bearing to left upper extremity, and follow up with orthopedic surgery in 4 weeks	Place in a sling, non-weight bearing to the left upper extremity, and follow up with PCP/sports medicine in 1 week	Place in coaptation splint, non-weight bearing to left upper extremity, and follow up with PCP/sports medicine in 1 week	Urgent Orthopedics consult for surgical intervention	
42. A football player presents to the ED with diffuse muscle pain after starting summer training camp 3 days ago. He notes tea-colored urine. What is the most likely finding on urinalysis?	(-)blood, full field RBCs, (+)myoglobin	(-)blood, 0 RBCs, (-)myoglobin	(+)blood, full-field RBCs, (+)myoglobin	(+)blood, 0 RBCs, (+)myoglobin	
43. A baseball player presents to the ED after sliding into a base and is unable to fully straighten his finger at the DIP joint. His x-ray is as shown (Figure 6). What is the most appropriate next step in management? Figure 6: Case courtesy of Andrew Taylor, Radiopaedia.org	Buddy tape and follow-up with ortho as outpatient	Closed reduction and splinting	Splint DIP joint in full extension	Urgent hand surgery consultation	Figure 6
44. You are evaluating a patient with shoulder pain after a motor vehicle crash. You suspect a clavicular injury and imaging is pending. What clavicular injury is most likely to have secondary life-threatening injuries?	Grade 2 Acromioclavicular joint dislocation	Midshaft clavicular fracture	Posterior sternoclavicular joint dislocation	Unstable distal clavicle fracture	
45. A patient presents to the ED with the following hand findings (Figure 7). What is the best treatment for this condition? Figure 7: Image courtesy of Adam Rosh, Rosh Review	Longitudinal incision along the radial aspect	Longitudinal incision over the pulp of the thumb	Oral antibiotics	Warm soaks and elevation	Figure 7
46. A 19-year-old college gymnast presents with left foot pain and swelling. He states he was tumbling and came down awkwardly on his foot. You are concerned for a Lisfranc injury. Which immobilization is most appropriate?	Compression bandage, non-weight bearing	Posterior long leg, weight bearing	Pneumatic boot, weight bearing	Post op shoe, non-weight bearing	
47. A 17-year-old patient dove into the shallow end of a pool, striking his head on the cement floor. He noted immediate midline neck pain but was neurovascularly intact. A CT scan of the cervical spine reveals a vertebral body fracture of C3. Which of the following suggests that this fracture is stable?	55% loss of vertebral body height	Minimal displacement of the posterior column	55% spinal canal narrowing	Posterior vertebral retropulsion	
48. An intoxicated patient is brought in by police to your ED after a fight with another bar patron. On exam, you note a deep laceration over the dorsal aspect of his 4th MCP joint, with concern for communication with the joint. He has normal ROM of his fingers. What is the most appropriate next step in management?	Bedside irrigation and closure	Hand surgery consultation	Leave open and place in an ulnar gutter splint	Leave open and discharge with oral antibiotics	
49. A 60-year-old male presents with left shoulder pain following a fall from standing height. He is neurovascularly intact and his trauma evaluation only demonstrates this isolated injury on radiograph (Figure 8). What is the optimal treatment for the represented injury? Figure 8: Case courtesy of Henry Knipe, Radiopaedia.org	Coaptation splint and urgent outpatient orthopedic follow-up	Shoulder immobilizer and emergent orthopedic consultation	Shoulder immobilizer and standard outpatient orthopedic follow-up	Shoulder sling and urgent outpatient orthopedic follow-up	Figure 8
50. A patient presents to the ED complaining of back pain. Which of the following is the most frequent cause of discitis?	Hematogenous spread	Localized spread	Recent surgery/procedure	Trauma	
